# Factors affecting interactome-based prediction of human genes associated with clinical signs

**DOI:** 10.1186/s12859-017-1754-1

**Published:** 2017-07-17

**Authors:** Sara González-Pérez, Florencio Pazos, Mónica Chagoyen

**Affiliations:** Computational Systems Biology Group, National Center for Biotechnology (CNB-CSIC), Darwin 3, 28049 Madrid, Spain

**Keywords:** Gene prioritization, Human interactome, Clinical signs, Network-based methods

## Abstract

**Background:**

Clinical signs are a fundamental aspect of human pathologies. While disease diagnosis is problematic or impossible in many cases, signs are easier to perceive and categorize. Clinical signs are increasingly used, together with molecular networks, to prioritize detected variants in clinical genomics pipelines, even if the patient is still undiagnosed. Here we analyze the ability of these network-based methods to predict genes that underlie clinical signs from the human interactome.

**Results:**

Our analysis reveals that these approaches can locate genes associated with clinical signs with variable performance that depends on the sign and associated disease. We analyzed several clinical and biological factors that explain these variable results, including number of genes involved (mono- vs. oligogenic diseases), mode of inheritance, type of clinical sign and gene product function.

**Conclusions:**

Our results indicate that the characteristics of the clinical signs and their related diseases should be considered for interpreting the results of network-prediction methods, such as those aimed at discovering disease-related genes and variants. These results are important due the increasing use of clinical signs as an alternative to diseases for studying the molecular basis of human pathologies.

**Electronic supplementary material:**

The online version of this article (doi:10.1186/s12859-017-1754-1) contains supplementary material, which is available to authorized users.

## Background

Clinical signs are manifestations of a patient’s underlying disease. While diseases are in many cases difficult or even impossible to diagnose, clinical signs are easier to recognize and in some cases, to quantify. It has recently been shown that clinical signs have a reflection at the molecular level; for example, their associated genes form modules in the interactome, at least to the same extent as diseases do [1]. All these factors make clinical signs a valid partition of the human pathological landscape, complementary to that based on diseases, which is receiving increasing attention.

In the study of the genetic basis of a disease, experimental validation of causal candidate genes is a time-consuming and expensive process. To save resources and maximize success, candidate genes obtained by genetic/genomic approaches can be prioritized with computational methods that consider a variety of previous information [2]. Among these, clinical signs of known genetic diseases can be compared to those manifested by a patient and be used to further prioritize variants obtained from whole exome analysis [3]. The obvious advantage of using patient’s clinical signs is that prioritization can be performed even if the patient has not yet been diagnosed or if his/her pathology is unknown. Some of these clinical genomics approaches, in addition to seeking whether a variant is already annotated with similar manifestations in human and other model organism databases, also examine molecular networks to prioritize variants following the guilt-by-association principle [4–6], as genes that cause the same or similar diseases are often found in close proximity in biological networks [7, 8].

Network-based methods used to prioritize candidate genes require an initial set of genes, referred to as ‘seed’ [9], typically comprised by those previously known to cause the diagnosed disease or causing diseases with similar clinical signs. Methods based on global distances, such as random-walk with restart (RWR) [10, 11], have been shown to outperform those based on local distances [12]. Several modifications of the RWR method have also been proposed to account for disease phenotypic similarities [13, 14]. The fact that disease genes map to relatively compact but not highly dense regions in the network of protein-protein interactions (interactome) have led to formulation of an alternative approach, Disease Module Detection (DIAMOnD) [15], which measures the connectivity significance of the candidates relative to known disease genes, instead of their distance.

**Fig. 1:**
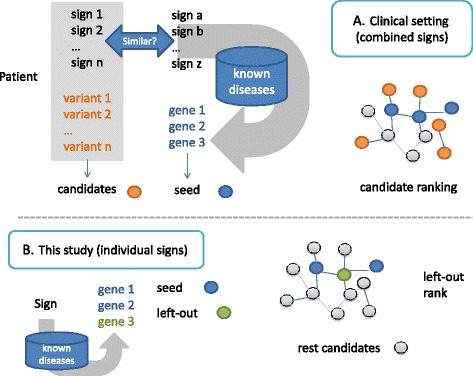
Comparison of a typical clinical setting and this study. **a** In a clinical setting patient’s clinical signs are combined to find similar known diseases and define seed genes. **b** In this study, genes associated to individual signs are predicted using a leave-one-out approach

In a clinical setting (Fig. 1a) candidate variants are first obtained by genetic analysis. Then all patient signs are combined to find known diseases with similar manifestations. The genes causing these similar diseases are then matched to the candidate variants. If no match is found, candidates can be prioritized using the interactome, using as seed those genes previously found to cause similar diseases. Thus the ability to successfully prioritize causal variants from both molecular networks and patient clinical signs depends, among other factors, on the ability of network methods to predict genes associated with these signs. Known gene associations to a sign have been used to predict novel genes associated to that particular sign using molecular networks [16]. We recently analyzed the network context of genes associated with clinical signs in the human interactome and observed that, as in the case of diseases, they generally form localized modules [1]. We nonetheless observed that the compactness of clinical signs varied notably, which is expected to affect prediction performance for different clinical signs.

In this study (Fig. 1b) we assessed the ability of network-based methods to predict genes associated with individual clinical signs. We compared two methods previously used to predict disease-gene relationships that follow two distinct approaches, random-walk with restart (RWR) [10, 11], based on global distances, and DIAMOnD [15], based on connectivity significance. While a conceptually similar approach to RWR, PRINCE [11], was previously used to predict sign-gene relationships [16], the ability of DIAMOnD to predict novel sign-genes has not been previously reported. We provide a detailed analysis of several clinical and biological factors that affect prediction performance. Our results point to several factors that should be taken into account when assessing the results provided by network-based approaches that use clinical signs and interactome data for gene prioritization, a practice in increasing use.

## Results

**Fig. 2:**
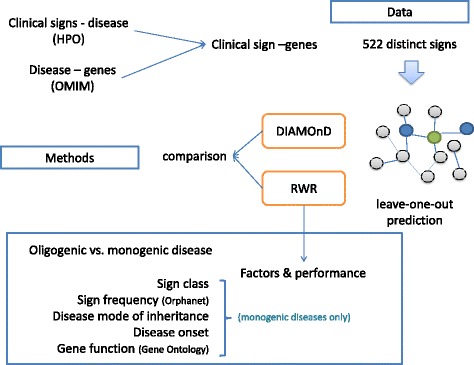
Overview of the data, methods and factors analyzed

An overview of the analysis performed is summarized in Fig. 2.

### Evaluation of network methods

To compare the performance of RWR and DIAMOnD, we tested 23,458 gene-clinical signs pairs from the OMIM and HPO databases, corresponding to 522 unique non-redundant signs and 2279 distinct genes (see Methods for details). In order to recreate a ‘de novo’ prediction scenario, for each prediction we removed one gene-clinical sign association (the one to be predicted) and used the remaining genes associated with the clinical sign as seed. As an example to illustrate our results we selected the clinical sign ‘Ketosis’, the presence of elevated levels of ketone bodies in the body, as defined in HPO. We found 29 diseases annotated with this sign in OMIM. Of these, 23 had at least one causal gene, providing a total of 28 genes associated with ‘Ketosis’. Three of these genes were not found in the interactome, thus leaving 25 genes for testing (from 19 distinct diseases). We then performed 25 predictions, one for each of these 25 genes, using the remaining 24 as seed. For each prediction we recorded the rank of the left-out gene among the remaining nodes in the interactome. Individual gene performances (using RWR) for this sign varied from best prediction (rank = 1) for IVD (isovaleryl-CoA dehydrogenase) causing isovaleric acidemia, to worst (rank = 7525) for GK (glycerol kinase) causing glycerol kinase deficiency. Overall RWR prediction performance for ‘Ketosis’, measured as the Area Under the ROC curve (AUC) of the 25 predictions, was 0.93.

**Fig. 3:**
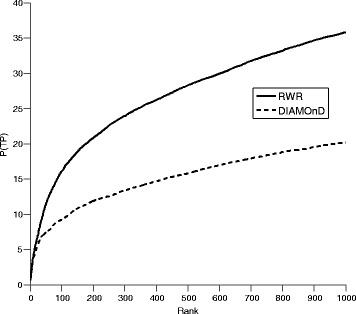
Performance of RWR and DIAMOnD methods. Performance is represented as percentage of true positives predicted among the top ranking genes (from rank 1 to 1000)

We found that RWR outperformed DIAMOnD on the 1–1000 rank range (corresponding to up to 1000 iterations of DIAMOnD) (Fig. 3). RWR predicted 35.85% of the genes among the top 1000, in contrast to 20.18% by DIAMOnD. Nonetheless, when we assessed performance for individual clinical signs (see Additional file 1: Table S1), DIAMOnD was able to capture more true positives in the top-ranking positions for a number of them. For example, within the top 100 (corresponding to a false positive rate (FPR) of 0.75%), DIAMOnD obtained better results than RWR for 60 of the 522 clinical signs. This number decreased at lower ranks, with 35 at rank = 500 (FPR = 3.75%) and 26 at rank = 1000 (FPR = 7.5%).

**Fig. 4:**
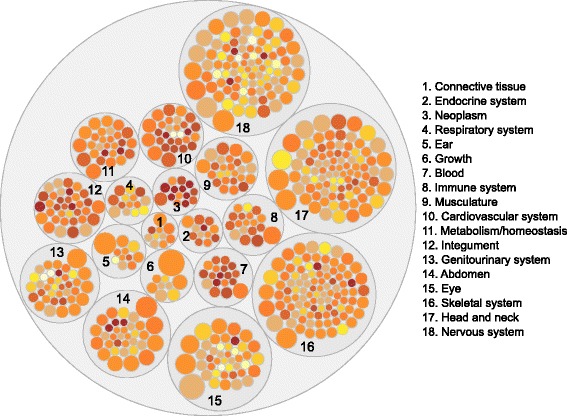
Variability in RWR performance for individual clinical signs. The 522 signs (small circles) are grouped into 18 general classes. Signs are colored by AUC value (darker for better performance). Circle size is proportional to number of genes

We observed high variability in the results for individual clinical signs for both RWR and DIAMOnD (see Fig. 4 for RWR results). Among the best RWR results in the top 100, we found ‘Cerebral edema’ with 85% of its 27 genes predicted, followed by ‘Lethargy’ (61%) and ‘Abnormality of the coagulation cascade’ (59%). In contrast, RWR was not able to predict any gene in the 100 top positions for 60 clinical signs. The best DIAMOnD results in the top100 were ‘Cerebral edema’ (52%), ‘Abnormal CSF lactate level’ (51%) and ‘Plantar hyperkeratosis’ (41%). DIAMOnD did not correctly predict any gene in the top 100 for 109 clinical signs. As RWR performed better overall than DIAMOnD, we used RWR for subsequent analyses.

### Analysis of clinical and biological factors that affect performance

Given the nature of the RWR algorithm, its performance depends on the compactness of each clinical sign in the interactome, which is variable [1]. The percentage of genes at distance 1 to another gene within the clinical sign in OMIM varies from 85% (cerebral edema) to 0% for 22 clinical signs, with an average value of 30% for all signs analyzed. As expected, there was a correlation between RWR performance and clinical sign compactness (not shown). We also evaluated the RWR prediction of 500 random sets (within variable random sizes in the 25–75 range) obtaining a performance very close to random prediction (AUC = 0.49).

The variability in compactness of clinical signs, and therefore prediction performance, can be explained by several clinical and biological factors. In this section we present a detailed analysis of some of these factors, including characteristics of the interactome, clinical sign type and frequency, monogenic/oligogenic nature of the associated disease, mode of inheritance and gene function.

### Compactness of biological processes in the interactome

Clinical signs can result from disruption of the same or different biological processes. As an upper bound of performance, we assessed the ability of RWR to predict genes involved in different molecular, cellular and organismal processes. We analyzed 1118 biological processes (distinct, non-redundant) from Gene Ontology.

We obtained better global performance for biological processes (AUC = 0.83) than for clinical signs (AUC = 0.70). In addition, RWR performance varied depending on the process, from best results for ‘DNA replication-dependent nucleosome organization’ (AUC = 1.0) to poorest for ‘cofactor transport’ (AUC = 0.42) (see Supplementary Additional file 2: Table S2).

Analysis of results per general classes of processes revealed that signaling and metabolic processes were among the best predicted, whereas growth, reproduction and developmental processes were among the poorest (Table 1). Given these results, we anticipated poorer performance for those clinical signs related, for example, to developmental processes than those related to metabolic processes. This later is the case of our illustrative sign ‘Ketosis’, a metabolic manifestation associated with several diseases, which achieved one of the best performances among the 522 signs analyzed (Supplementary Additional file 1: Table S1). As expected, prediction performance for multicellular organismal processes (AUC = 0.78) was lower than for cellular processes (AUC = 0.86).

**Table 1:** RWR performance for gene-process prediction, according to general GO class

GO class	N° proc.	AUC(%)
signaling	69	89.50
metabolic process	196	88.27
response to stimulus	177	85.66
cellular process	461	85.58
immune system process	46	85.54
cellular component organization or biogenesis	100	85.18
biological regulation	519	82.79
single-organism process	518	82.65
multi-organism process	22	82.37
locomotion	14	82.12
localization	89	82.05
biological adhesion	8	80.76
multicellular organismal process	190	77.68
reproductive process	14	77.58
developmental process	172	77.03
reproduction	10	76.13
growth	5	72.85

### Monogenic vs. oligogenic diseases

In the case of oligogenic diseases, we can use their known genes to predict others. When predicting genes associated with monogenic diseases no other disease genes are known and, as in the case of undiagnosed patients, indirect strategies are needed to build the starting set of genes (seed). One of these strategies, used in some genomic pipelines [4–6], is to build a seed with genes of diseases with similar clinical manifestations. Clinical signs might thus seem most valuable in the discovery of variants underlying monogenic diseases or generally in those cases where a patient is still undiagnosed or his/her disease is unknown.

In this work, we defined a disease as a distinct OMIM phenotypic code, and classified them as mono- or oligogenic based on the number of associated genes in the OMIM database that could be mapped in the interactome (1 for monogenic, >1 for oligogenic; individual OMIM phenotypes in phenotypic series were not treated as oligogenic disease). For example, among the 19 diseases with ‘Ketosis’ as clinical sign, two of them were classified as oligogenic: maple syrup urine disease with 3 genes (BCKDHA, BCKDHB and DBT) and Permanent neonatal diabetes mellitus with 4 genes (GCK, INS, KCNJ11 and ABCC8). The remaining 17 diseases were classified as monogenic.

In this section we analyzed separately those ranks obtained for genes associated with oligogenic diseases (including 7 genes for ‘Ketosis’) and those for monogenic diseases (including 17 genes for ‘Ketosis’). In terms of gene-sign pairs, 19,973 corresponded to monogenic and 3894 to oligogenic diseases (409 pairs are from both mono- and oligogenic diseases), for a total of 3060 monogenic and 170 oligogenic diseases. Note that when predicting genes for a sign of a monogenic disease, seed genes come necessarily only from other diseases with that specific sign. In contrast, when predicting a gene for a sign associated with an oligogenic disease, seed genes contain genes from other diseases as well as other genes affecting that particular disease.

The performance of RWR in predicting genes associated with clinical signs of oligogenic diseases (AUC = 0.84) was notably better than for those of monogenic diseases (AUC = 0.68). As (i) these results might affect the analysis of other factors, (ii) monogenic diseases are more numerous in our dataset than oligogenic diseases, and (iii) it is possible to directly predict genes associated with oligogenic diseases using other known genes as seed, we analyzed other factors affecting performance only for monogenic diseases. In the ‘Ketosis’ example, 7 out of 25 genes came from oligogenic diseases, thus only the ranks of the remaining 17 were considered in the following sections.

The alternative direct prediction of genes for oligogenic diseases with RWR (e.g. predicting DBT gene as associated to maple syrup urine disease using only BCKDHA and BCKDHB genes as seed) yielded an AUC of 0.85. This is only slightly better than the overall prediction obtained through clinical signs (AUC = 0.84). To test whether particular clinical signs could improve the ranking obtained by direct predictions of genes for oligogenic diseases, we extracted the best rank for each gene from among all signs for each disease. If we knew the best clinical sign for each gene beforehand (not known a priori), this would give us an upper bound of AUC = 0.91.

### Clinical sign frequency

Some clinical signs are frequent manifestations of certain diseases, whereas others appear less frequently. It is reasonable to expect that those genes causing diseases for which the sign is less frequent (low prevalence) are predicted more poorly than those for which the sign is very frequent (high prevalence). We therefore compared the performance of gene-signs predictions for monogenic diseases compiled in Orphanet, for which sign frequency data is available. In this resource, signs are classified as ‘hallmark’, ‘typical’ and ‘occasional’ on a decreasing scale of prevalence.

As expected, hallmark signs had slightly better overall performance (AUC = 0.70) than typical (AUC = 0.69) and occasional signs (0.68), although differences are only marginal.

### Mode of inheritance

Genes associated with autosomal dominant diseases are found mainly as hubs or bottlenecks in the interactome, whereas those associated with autosomal recessive diseases are frequently located at the periphery [17]. The inheritance pattern of the disease(s) associated with a given clinical sign is thus expected to affect the ability of network-based methods to predict genes associated with it. In this section we analyzed separately the ranks of gene-sign predictions corresponding to ‘autosomal dominant’ and ‘autosomal recessive’ diseases. Indeed, RWR rendered a prediction performance of AUC = 0.78 for the 8116 gene-sign pairs associated with monogenic diseases annotated as ‘autosomal dominant’, and a much lower value for the 12,558 gene-sign pairs of ‘autosomal recessive’ monogenic diseases (AUC = 0.63).

In the example case, ‘Ketosis’, 14 of the 17 monogenic diseases were annotated as ‘autosomal recessive inheritance’, so the ranks of the 14 genes associated with these diseases were analyzed as part of the autosomal recessive group. Among the 17 ketosis-associated monogenic diseases, no monogenic autosomal dominant diseases were found, so this sign did not contribute to the analysis of autosomal recessive diseases.

### Clinical sign classes

The variability of results for different clinical signs is also expected to be shown at broad sign classes. We analyzed the ranks obtained by RWR (monogenic diseases only) grouped by 18 classes of clinical signs, corresponding to the most general HPO terms under ‘Phenotypic abnormality’ (note that a given sign can belong to various classes). Performance varied among clinical sign classes (Table 2). The best results were obtained for ‘neoplasms’ and signs related to blood and blood-forming tissues. Signs of the endocrine, cardiovascular, immune systems, integument and metabolism/homeostasis classes had a performance score of AUC > 0.7. RWR generally performed more poorly for signs related to the eye, nervous system, growth abnormalities and ear. Our ability to predict genes thus currently depends on the type of clinical signs a patient manifests.

**Table 2:** RWR performance for gene-sign prediction, according to general HPO classes

Clinical sign class	N° pairs	AUC (%)
Neoplasm	235	80.77
Abnormality of blood and blood-forming tissues	566	77.02
Abnormality of the endocrine system	307	74.56
Abnormality of the cardiovascular system	1027	73.84
Abnormality of the immune system	609	72.91
Abnormality of the integument	1155	72.68
Abnormality of metabolism/homeostasis	953	72.15
Abnormality of the musculature	895	69.47
Abnormality of the abdomen	1444	68.45
Abnormality of connective tissue	293	67.99
Abnormality of the skeletal system	3460	67.52
Abnormality of head and neck	4071	67.33
Abnormality of the respiratory system	406	67.17
Abnormality of the genitourinary system	1182	66.55
Abnormality of the ear	628	65.74
Growth abnormality	547	65.71
Abnormality of the nervous system	3995	64.70
Abnormality of the eye	1908	64.39

‘Ketosis’, our example sign, is classified according to HPO as an ‘Abnormality of metabolism/homeostasis’. To measure the overall performance this group of signs, we analyzed all the ranks obtained for genes causing monogenic diseases (including the 17 genes associated with ‘Ketosis’), together with those genes associated with other metabolism/homeostasis signs. The overall performance of this general class of signs was AUC = 0.72.

### Disease onset and pace of progression

Severity of manifestations in the organism can vary within the interactome throughout life. We therefore anticipated some differences at the interactome level between diseases that arise early in development and those that appear later, as well as between those with slow versus rapid progression, which in turn would affect the performance of network-based methods for predicting genes associated with clinical signs. We analyzed the ranks of gene-sign predictions grouped by disease onset according to six HPO annotations: congenital (at birth), neonatal (first 28 days), infantile (28 days-1 year), childhood (1–5 years), juvenile (5–15 years) and adult (>15 years). In general, prediction performance increases from earlier to later age (Table 3), except for ‘juvenile’ and ‘neonatal’ onsets. In the Ketosis example, only two diseases were associated with onset data: Methylmalonic aciduria, cblA type (caused by MMAA) annotated as both ‘neonatal’ and ‘infantile’ and Glycogen storage disease IXc (caused by PHKG2) which also an infantile disease according to HPO. Therefore the ranks obtained for the Ketosis predictions of MMAA (rank = 15) and PHKG2 (rank = 604) were used, among other signs, to calculate the overall performance of infantile onset, while only the Ketosis prediction for MMAA contributed (together with other sign results) to the performance of neonatal onset.

**Table 3:** RWR performance for gene-sign prediction, according to disease onset

Onset	N° pairs	AUC(%)
Juvenile onset	993	75.08
Neonatal onset	203	74.93
Adult onset	1068	72.80
Infantile onset	2410	70.13
Childhood onset	341	69.03
Congenital onset	1518	65.32

Similarly, predictions are better for signs associated with non-progressive disorders (AUC 0.79) than for progressing pathologies (AUC 0.61) (see Table 4 for details).

**Table 4:** RWR performance for gene-sign prediction according to disease pace of progression

Pace of progression	N° pairs	AUC (%)
Nonprogressive disorder	219	78.68
Slow progression	1112	65.79
Progressive disorder	1657	61.24
Rapidly progressive	359	60.30

### Gene product function

Protein function can affect prediction performance in two ways; 1) due to the way it was constructed, our interactome might be enriched in certain functions to the detriment of others and, as shown above, 2) the interactome compactness of different biological processes affects the results as well. We analyzed the differences in performance for three aspects of protein function, molecular activity (MF), biological processes (BP) and cellular localization (CC) using its Gene Ontology annotations (see Additional file 3: Table S3).

We evaluated the overall performance of a GO term by calculating the AUC values from the ranks of those gene-sign pairs whose genes were annotated with that particular term. For example, 20 the 21 genes of monogenic diseases associated to ‘Ketosis’ (all except MMAA gene) were annotated with ‘metabolic process’ term in GO. So their corresponding ranks (obtained for Ketosis prediction), as well as the ranks of all gene-sign pairs corresponding to ‘metabolic process’ genes, were used to calculate the overall ‘metabolic process’ performance (AUC = 0.69).

Among the results with the highest performance for molecular activities were ‘chemoattractant’, ‘proteasome regulator’, ‘translation regulator’ and ‘antioxidant’, with above average performance for ‘structural molecule’, ‘molecular transducer’ and ‘transcription regulator’, and below average for unknown, ‘metallochaperone’, ‘transporter’ and ‘nutrient reservoir’. In the case of biological processes, we observed better performances for ‘reproduction’, ‘cell killing’ and ‘biological adhesion’, below average performances for unknown, ‘viral reproduction’, ‘immune system process’, ‘growth’ and ‘response to stimulus’. Finally, highest AUC values for cellular localization were ‘chromosomal part’, ‘extracellular matrix’, ‘cytoplasmic vesicle’ and ‘nuclear part’, whereas the lowest and below average values were for ‘mitochondrial membrane’, unknown, ‘endosomal part’, ‘endoplasmic reticulum’ and ‘cilium’.

### Impact of factors on a clinical setting

In the previous sections, we analyzed the impact of several clinical and biological factors on the network-based prediction of genes associated to individual clinical signs. In a clinical setting (Fig. 1a), however, network-based methods consider simultaneously all of a patient’s clinical manifestations, not each individual sign in isolation. We anticipate that the factors analyzed for individual signs in this study would also have an impact in this use-case (disease-gene prediction).

To confirm this, we analyzed the impact of a subset of factors on disease-gene prediction. For this analysis we performed a leave-one-out prediction of gene-disease pairs, instead of gene-sign pairs. For example, to predict the association of IVD gene to isovaleric academia, a monogenic disease according to OMIM, we first obtained the disease’s signs (ketoacidosis, among others), including also their ancestor terms in the HPO hierarchy. We then searched for diseases manifesting at least one of those clinical signs, and compiled their associated genes. These genes (excluding IVD) were used as seed for RWR prediction (see Methods for details on seed weights). Seed genes in this case, are therefore obtained from the combination of all clinical signs associated to a disease.

Oligogenic diseases were better predicted than monogenic (as for individual signs). Analysis of monogenic diseases showed that overall disease-gene prediction performance (AUC 0.79) was higher than overall clinical sign-gene prediction (AUC 0.68). Nevertheless, variability and trends for each factor analyzed in the two scenarios were generally similar (see Additional file 4: Table S4).

## Discussion

Clinical signs associated with genetic diseases, together with molecular networks, are being used increasingly in clinical genomics pipelines to help prioritize genomic variants [4–6]. Although clinical signs map to localized areas in the currently known human interactome, their compactness varies notably [1] and can thus have a different effect on gene prioritization performance.

Using 23,458 gene-clinical signs pairs compiled from OMIM and HPO databases and the same human interactome data analyzed in our aforementioned study of compactness [1], we compared two network approaches used previously for associating genes to diseases for their capacity to predict associations between genes and signs. These are based on global network distances (RWR) and connectivity significance (DIAMOnD). RWR outperformed DIAMOnD on overall prediction results. DIAMOnD was nonetheless reported to perform better than RWR in lower ranks in the analysis of synthetic modules [15]. Our results suggest that global distances are generally more informative than connectivity significance when predicting genes associated with clinical signs. DIAMOnD nonetheless provided better results than RWR at lower ranks for some of the clinical signs tested. Connectivity significance is therefore especially valuable when no direct interactions are available.

Prediction performance varied for different clinical signs. Similarly variable performance is also described for predicting disease-associated genes [12]. We can explain this by the different degree to which clinical signs are reflected as compact modules in the interactome, which in turn depends on a number of clinical and biological factors analyzed. All these factors are expected to have an impact in the network-based prioritization of variants for unknown diseases and undiagnosed patients, as they account for general trends observed in the analysis of already known diseases.

Molecular networks reflect different functional modules in the cell, such as molecular complexes and biological processes [18]. The molecular network analyzed here mainly represents direct physical interactions between proteins, and a smaller set of other types of functional relations. We assessed the ability of RWR to recover genes that participate in different biological processes. RWR was able to predict genes associated with biological processes with good performance. Performance nonetheless varied from one process to another, with poorer results in general for multicellular organismal processes than for cellular processes. This is reasonable, as multi-cellular processes must be orchestrated at higher levels that are probably beyond protein interactions.

Genes associated with clinical signs of oligogenic diseases can be prioritized using previously known disease-genes as seeds. For oligogenic diseases, we observed similar performance by this direct disease-gene prediction as that of overall sign-gene prediction. Direct disease-gene prediction is not possible for monogenic diseases, however, and alternative strategies must be used to build a seed, like those based on diseases with similar clinical signs [4–6]. We obtained notably better results predicting gene-sign pairs for oligogenic than for monogenic diseases. Our results suggest that genes from oligogenic diseases are involved in closer molecular mechanisms than genes from distinct monogenic diseases, even when they manifest the same clinical sign. We analyzed other factors affecting performance only for monogenic diseases as they recreating the case of undiagnosed patients or those manifesting diseases with unknown etiology.

Mode of inheritance is another important factor to consider. Genes associated with signs of autosomal dominant diseases (AD) were better predicted than those corresponding to autosomal recessive diseases (AR). Our observations agree with a previous study that observed that AD disease-genes tend to be hubs or bottleneck genes on the interactome, whereas AR disease-genes were found mostly in the periphery [17].

Prediction performance was also variable for clinical sign classes. RWR generally performed more poorly for signs related to the eye, nervous system, growth abnormalities and ear. Some of these were also reflected in the analysis of biological processes. This might be due to inherent differences in the network topology of these processes, or to our still incomplete interactome. If the latter is the case, our results highlight relevant clinical manifestations that are currently under-investigated at the molecular/network level, and hence point to underexplored interactome regions that merit further attention.

Gene function might affect interactome completeness and explain variable performance. Genes with unknown functions performed less well than genes with known functions (in all types of functions), even if functional annotations were not explicitly considered during prediction. This can be explained by the interactome analyzed, for which almost half of the interactions are derived from “literature-curated” low-throughput experiments, typically performed on well-studied proteins. For a small percentage of these ‘unknown function’ genes, we were nonetheless able to predict a clinical manifestation.

Genes acting as nutrient reservoirs, metallochaperones and transporters were predicted below average. This could be due not to direct, but to indirect links (through nutrients, metal ions and substances transported) not reflected in our interactome. Similar poorer-than-average results were obtained for genes involved in processes related to ‘viral reproduction’, ‘immune system process’, ‘growth’ and ‘response to stimulus’, again probably due to systemic or environmental factors not reflected in the interactome. Those genes located in the mitochondria, endosomes, endoplasmic reticulum, Golgi apparatus and cilia were also generally poor performers. All of these locations can affect the overall energy flow and molecular content of the cell by mechanisms not involving direct protein interactions. Genes involved in reproduction were among the best for predicting their association to clinical signs, but were among the poorest when predicting their association to their own processes (‘reproductive process’, as defined in the Gene Ontology).

Better predictions were obtained for signs associated with diseases with early onset. This could be due to the greater severity of these diseases, which could in turn be related to more compact modules in the interactome. Finally, the frequency with which a clinical sign manifests in a disease affected the prediction results only marginally. In any case, frequency data are currently available only for a subset of diseases. It would be of interest to collect and use this type of information and further consider it in the analysis.

All of these factors would also affect network-based disease-gene prediction approaches, in which the entire spectrum of signs manifested in a patient are considered simultaneously. Here we analyzed a number of such factors and observed a trend similar to the case of individual sign prediction.

Our current ability to predict genes associated with clinical signs is quite variable, and is related to their topology in the human interactome. This variable topology can be explained by a partially incomplete interactome, but can also reflect the different natures of the molecular mechanisms that underlie clinical signs. Prediction performance will improve as our knowledge of the human interactome completes and as new associations between genes and clinical signs are compiled. Therefore, in a clinical setting it will advantageous to integrate data from multiple sources to collect the most updated and complete human interactome and reference set of known gene-sign associations. We nonetheless suspect that network approaches such as RWR will never reveal the more ‘indirect’ functional linkages inherent to the nature of some clinical signs, especially those involved in multicellular processes. A deeper analysis of these functional linkages will certainly help to develop novel strategies and approaches, and therefore improvements on current prediction performance.

## Conclusions

Our results show that global network distance has greater predictive value than connectivity significance for prediction of genes associated with individual clinical signs. Performance is extremely variable, and depends essentially on the topology of the sign in the molecular network, which is in turn affected by different clinical and biological factors. In practical terms, our recommendation is that the characteristics of clinical signs and their related diseases be taken into account for interpreting the results of network-prediction methods.

A partition of the human pathological landscape in clinical signs has obvious advantages, since these are easier to identify and classify than diseases. As a consequence, signs are being used increasingly to study the molecular basis of human pathologies. Our results point to important factors that should be taken into account in such studies.

## Methods

### Data

Clinical sign-gene associations were compiled as in a previous study [1]. Briefly, diseases and their clinical signs and symptoms were downloaded from the Human Phenotype Ontology (HPO) [19] and disease-gene associations were obtained for OMIM (Online Mendelian Inheritance in Man) [20], as provided by the HPO. As our objective was to assess the impact of clinical and biological factors on collection of non-redundant diseases we chose OMIM as a public and carefully curated database of genetic diseases and HPO as the source of human clinical manifestations that is used in current genomic pipelines that integrate network and clinical signs. Clinical sign-gene pairs were generated based on their associations with the same disease(s). These sign-gene associations were further expanded to ancestor terms in the HPO hierarchy. We then selected those terms with at least 25 genes in the ‘Phenotypic abnormality’ sub-ontology, and kept only the most specific terms among them according to the HPO hierarchy for further analysis. This resulted in 522 unique non-redundant signs. In this way, we avoided redundancy, while a minimum size of 25 genes allows the detection of meaningful modules in the interactome analyzed [21].

Interactome data were obtained from the supplementary data of [21]. These authors compiled an integrated human interactome of 141,296 physical interactions among 13,460 proteins, comprising literature-curated interactions, high-throughput physical interactions, protein complexes, regulatory interactions, enzyme-coupled interactions, kinase-substrate pairs and other signaling interactions. We evaluated the performance of predictions only for those genes associated with clinical signs available in the interactome. The final test set comprised a total of 23,458 clinical sign-gene pairs, corresponding to 522 distinct signs and 2279 distinct genes.

Data on mode of inheritance of diseases, their onset and pace of progression was obtained from HPO. Prevalence data on clinical signs (their frequency in their respective diseases) was obtained from Orphanet diseases (Orphanet: an online rare disease and orphan drug data base. © INSERM 1997. Available on http://www.orpha.net). Frequency data of clinical signs for each disease were transferred to the corresponding sign-gene pair. Using the same approach described above for OMIM diseases, we analyzed a final set of 436 non-redundant specific clinical signs with at least 25 genes for Orphanet diseases. Gene Ontology annotations were downloaded from DAVID gene (Database for Annotation, Visualization and Integrated Discovery) [22].

### Prediction analysis

Using this set of clinical-sign-gene pairs we then performed a leave-one-out cross-validation with both RWR and DIAMOnD algorithms; for each clinical sign we removed one gene at a time (gi), and made a prediction for it using the remaining genes as seed, with equal probabilities. We then assessed the rank assigned to each test gene (gi). The rank for DIAMOnD corresponds to the iteration in which the test gene is added to the seed. A rank of 1 would thus mean that the gene was found in the first iteration of the method. For RWR, we obtained a ranked list of genes, with all the nodes in the interactome sorted by final score. The rank assigned to test gene gi is the position in this sorted list, excluding the seed genes. The gamma parameter for RWR was set to 0.4, since that value yielded the maximum overall performance in our tests.

We used the MATLAB implementation of RWR distributed by [14]. The adjacency matrix constructed from the human interactome was normalized as in PRINCE (Prioritization and Complex Elucidation) [11]. We used the DIAMOnD algorithm [15] available at https://github.com/barabasilab/DIAMOnD.

For disease-gene prediction, we used the RWR method only, performing a leave-one-out cross-validation. We selected those OMIM diseases with at least one gene and one phenotypic abnormality in HPO. For each disease-gene pair, genes associated with all the HPO terms annotated for the test disease and their parents in the ontology (except the one to predict) were used as seeds. Initial probability of each seed gene was set to max[1/(ni-1)], where ni is the number of genes associated with term i, for all its associated HPO terms.

## Additional files

Additional file 1: Table S1. RWR and DIAMOnD results for the individual clinical signs tested (XLSX 62 kb)

Additional file 2: Table S2. RWR performance for GO biological process prediction (XLSX 47 kb)

Additional file 3: Table S3. RWR performance according to gene function. Functions correspond to Gene Ontology molecular function (MF), biological process (BP) and cellular component (CC). (XLSX 11 kb)

Additional file 4: **Table S4.** Impact of factors on disease-gene prediction. (XLSX 11 kb)
